# Signatures of the collapse and incipient recovery of an overexploited marine ecosystem

**DOI:** 10.1098/rsos.170215

**Published:** 2017-07-05

**Authors:** Eric J. Pedersen, Patrick L. Thompson, R. Aaron Ball, Marie-Josée Fortin, Tarik C. Gouhier, Heike Link, Charlotte Moritz, Hedvig Nenzen, Ryan R. E. Stanley, Zofia E. Taranu, Andrew Gonzalez, Frédéric Guichard, Pierre Pepin

**Affiliations:** 1 Department of Biology, McGill University, Montreal, Quebec, Canada; 2 Center for Limnology, University of Wisconsin-Madison, Madison, WI, USA; 3 Department of Ecology and Evolutionary Biology, University of Toronto, Toronto, Ontario, Canada; 4 Marine Science Center, Northeastern University, Nahant, MA, USA; 5 Institute for Ecosystem Research, Kiel University, Kiel, Germany; 6 PSL Research University: EPHE-UPVD-CNRS, USR 3278 CRIOBE, BP 1013 Papetoai, 98729 Moorea, French Polynesia; 7 Laboratoire d'Excellence ‘CORAIL’, Guadeloupe, France; 8 Département des sciences biologiques, Université du Québec a Montréal, Montréal, Quebec, Canada; 9 Fisheries and Oceans Canada, Bedford Institute of Oceanography, Dartmouth, Nova Scotia, Canada; 10 Département des sciences biologiques, Université de Montréal, Montréal, Quebec, Canada; 11 Fisheries and Oceans Canada, Northwest Atlantic Fisheries Centre, St John's, Newfoundland and Labrador, Canada

**Keywords:** community ecology, ecosystem-based management, community synchrony, spatial ecology, regime shifts, marine ecology

## Abstract

The Northwest Atlantic cod stocks collapsed in the early 1990s and have yet to recover, despite the subsequent establishment of a continuing fishing moratorium. Efforts to understand the collapse and lack of recovery have so far focused mainly on the dynamics of commercially harvested species. Here, we use data from a 33-year scientific trawl survey to determine to which degree the signatures of the collapse and recovery of the cod are apparent in the spatial and temporal dynamics of the broader groundfish community. Over this 33-year period, the groundfish community experienced four phases of change: (i) a period of rapid, synchronous biomass collapse in most species, (ii) followed by a regime shift in community composition with a concomitant loss of functional diversity, (iii) followed in turn by periods of slow compositional recovery, and (iv) slow biomass growth. Our results demonstrate how a community-wide perspective can reveal new aspects of the dynamics of collapse and recovery unavailable from the analysis of individual species or a combination of a small number of species. Overall, we found evidence that such community-level signals should be useful for designing more effective management strategies to ensure the persistence of exploited marine ecosystems.

## 1. Introduction

Overexploitation has led to the collapse of many of the world's fisheries [1]. Although many fisheries have successfully recovered following reductions in fishing pressure [2], recovery rates are often slow and unpredictable [3,4]. Further, large-scale changes in the ecological community a fishery is embedded in may prevent ecosystems from recovering back to their former composition [5]. Sustainable fisheries management requires a better understanding of the health of marine ecosystems, and indicators that can allow us to detect when changes are occurring. Ecologists and managers responsible for keeping track of marine fish populations have historically focused on the population dynamics of one or a few commercially important species, paying little attention to the dynamics of connected predator, competitor and prey species [6]. These approaches have provided deep insights into the dynamics of commercially important species [7] and, when applied effectively, have led to successful stock recoveries. However, single-species management has not always been effective at predicting recovery rates [4], and has failed to restore stock biomass in some major fisheries [8,9].

The most iconic case of an unrecovered fishery is the North Atlantic cod stock (*Gadus morhua*), which collapsed in the early 1990s after decades of overfishing and an extended period of cold surface water temperatures [3,10,11]. In 1992, a fishing moratorium was imposed to allow the stocks to recover and was initially intended to last for 2 years [12]. However, cod did not recover, and the directed fishery remains closed [13]. The recovery of the Northwest Atlantic cod stock has been far slower than expected [14], even when accounting for the species' slow maturation rate and high trophic level [3,15].

Prior to the collapse, cod was the dominant large-bodied predator in the groundfish community [15]. Evidence suggests that the dramatic decline of cod was associated with that of other groundfish species, and these collapses in turn triggered a trophic cascade, resulting in an increase in the biomass of large benthic invertebrates [16]. Changes in the groundfish community also coincided with distinct alterations in forage fish across ecosystems, with capelin (*Mallotus villosus*) collapsing abruptly in Newfoundland [17], but increasing on the southeastern Scotian shelf [18,19].

To date, efforts to understand the collapse and lack of recovery have largely focused on cod-specific responses to environmental drivers such as temperature or residual fishing mortality [20]. However, on the Scotian shelf, initial signs of recovery are evident in other species: declines in forage fish have reduced predation pressure on juvenile cod and predator biomass has increased since 2005 [21]. By contrast, on the Newfoundland shelf, cod recovery appears to be limited by the lack of forage fish [22]. Still, recent stock assessments report modest increases in cod biomass on the Newfoundland shelf [13,23] and the Grand Banks [24], suggesting that the ecosystem may be starting to recover. The interdependence of cod with other species suggests that the collapse and recovery of this ecosystem may be better understood by studying the groundfish community as a whole [24].

Single-species monitoring is most likely to fare poorly in cases where the broader ecological community is changing rapidly [9]. These changes may either be due to shifts in drivers exogenous to a given species, such as large-scale changes in productivity [25], or occur when harvesting drives changes in the composition of the community, such as trophic cascades caused by overharvesting of a top predator [19]. We refer to such large-scale ecological changes as regime shifts, regardless of whether they are due to exogenous or endogenous causes. Regime shifts are increasingly recognized as an important factor in marine management [26], but we still lack a coherent set of tools to identify when they are occurring in multi-species systems.

Information gleaned from the collective response of multiple species to overfishing and environmental change can capture signatures of changing ecological regimes that are not apparent from the dynamics of individual species. Fishing and other stressors can substantially shape benthic ecosystems, from direct impacts of gear on benthic habitat and species abundances [27,28] to cascading impacts that lead to large-scale shifts in community structure [29,30]. However, the dynamics of community-level indicators are still only rarely used to inform fisheries management. Here, we focus on five key indicators that reveal complementary information about the community dynamics: community abundance, community synchrony, community composition, functional diversity and spatial community structure. Increasing temporal synchrony in the abundances of different species can reveal common responses to drivers, and may indicate that the entire community is undergoing a regime shift [31]. Likewise, shifts in the relative abundance of species in space and time can indicate changing biotic interactions or abiotic conditions, implying that community dissimilarity from a baseline state should increase during regime shifts, and decrease if the system returns to its previous state [24,32–35]. The functional consequences of these compositional shifts are reflected in traits of the community and these changes can be quantified using measures of functional diversity [36,37]. The functional consequence of regime shifts in community composition can be buffered if species are functionally redundant, and a return to previous functional diversity may indicate ecosystem recovery. Furthermore, large-scale changes in spatial community structure can lead to changes in metacommunity stability and resilience [38], and may indicate impending regime shifts [39,40]. Regime shifts do not always occur simultaneously across entire regions, but may instead start in localized areas [41,42], which can only be detected by tracking spatial changes in community composition. Community properties such as biomass synchrony, functional diversity and composition can respond to external drivers over different temporal and spatial scales [43], and collectively, provide a more complete picture of the health of the ecosystem.

As one of the most well-known and well-studied examples of a marine regime shift, the collapse of the Newfoundland shelf cod stock (and associated groundfish community) is an ideal case study for determining how different community indicators can be used to understand regime shifts. We analysed the collapse of the Newfoundland groundfish community and identified signs of the ongoing recovery, using data from the Canadian Department of Fisheries and Oceans autumn multi-species trawl survey. We demonstrated how different aspects of the groundfish community (biomass, synchrony, composition, functional diversity and spatial structure) collapsed and recovered at different rates, and how the temporal dynamics of these different community properties collectively provide novel insights into the ongoing recovery of this exploited ecosystem.

## 2. Material and methods

Our primary goal here was to test how different indicators of community structure changed throughout the collapse and the beginning of recovery of the Newfoundland shelf groundfish community. Each indicator chosen was readily calculable from spatial community abundance data (biomass), commonly collected in many large-scale marine fisheries, and functional trait information, which is increasingly available through public databases (e.g. fishbase.org) and the scientific literature. We tracked changes in each indicator prior to and after the two large regime shifts observed in this system: the collapse of cod stocks in the early 1990s and the early stages of potential recovery of cod in the last 5–10 years. All analyses were applied to two subsets of the community: the whole groundfish community and the groundfish community excluding the four commercially dominant species (Atlantic cod (*G. morhua*), Greenland halibut (*Reinhardtius hippoglossoides*), American plaice (*Hippoglossoides platessoides*) and deepwater redfish (*Sebastes mentella*)), to determine if changes in the community indicator were being driven solely by the decline of commercial stocks or if they indicated a broader change in the shelf community.

The data consisted of the recorded abundance, measured as biomass per trawl, of all sampled groundfish from an average of 421 (s.d. = 62) net trawl hauls per year (14 688 samples). The survey used a random depth-stratified sampling design, with fixed duration and speed trawls. From 1978 to 1994, trawls were conducted at 3.5 knots for 30 min, using an Engel otter trawl. In 1995, trawls switched to using a Campelen shrimp trawl, with smaller mesh size, and conducted at 3 knots for 15 min, to account for the larger catches of the Campelen trawl. While trawls were not standardized for area swept by a given tow, any trawl that was substantially shorter than planned (20 min or less for Engel trawls, 10 min or less for Campelen trawls) was discarded [44].

This study used the portion of the dataset spanning the North Atlantic Fishing Organization (NAFO) divisions 2 J, 3 K and 3 L, off the coast of Newfoundland, Canada, excluding inshore trawls (electronic supplementary material, figure S1). We used data spanning the period from 1981 to 2013. Years prior to 1981 (1968–1980) were excluded because samples did not cover the entire study region [44].

The change in gear from Engel to Campelen meant that more small fish were being caught from 1995 onward, which could potentially bias results as it would increase measured biomass and the probability of observing small species after 1994. While conversion factors have been calculated for a few species [45], they do not exist for most, and no multiplicative factor could account for species absences prior to the gear change. To mitigate this bias, we screened out species that were captured infrequently prior to the gear change (such as invertebrates) or were particularly sensitive to the gear change, defined as any species whose mean biomass between 1995 and 2000 was more than seven times greater than the mean pre-1995 biomass. The final dataset included 30 fish species (electronic supplementary material, table S2). To determine how sensitive our results were to the gear change, we also estimated conversion factors by matching trawls from before and after the change, and calculating the mean ratio of biomass between matched trawls for each species, using a random effects model (details in electronic supplementary material). However, as the estimates for conversion factors were highly variable and our conclusions did not substantially change with the inclusion of conversion factors, we used the untransformed values for the analyses in the main text. To aid the reader in identifying changes in the indicators driven by the gear change, we divided time series into before and after gear change in all figures.

### 2.1. Constructing spatial grid of study area

To develop a set of consistently spatially sampled subareas, we divided the study area into a set of Voronoi polygons [46] using the *inla.mesh.2d* mesh algorithm from the R INLA package [47]. The constraints on the algorithm were selected such that the majority of polygons included one or more trawl locations per year, and that the range of trawls per polygon per year was consistent. This resulted in 150 unique polygons, with an average of 1–6.3 trawls per year (2.5–4.0 trawls per year interquartile range) for the years in which a trawl occurred in that polygon (not every polygon had data present in every year). We calculated the average observed depths in each polygon as the arithmetic mean of the observed depths recorded for all trawls in that polygon.

### 2.2. External drivers

We calculated time series of exogenous large-scale drivers to determine how closely our community-level metrics tracked larger external ecosystem changes. We calculated fishing effort from NAFO data (series 21B; www.nafo.int/Data/Catch-Statistics) based on time spent at sea by fishing vessels in the NAFO divisions 2 J, 3 K and 3 L; we first identified all ships recorded as targeting benthic fish, then multiplied the days at sea for each vessel by the tonnage of that vessel, and summed values within years. This yielded an estimate of fishing effort in each year, measured in tonne-days.

To represent large-scale climatic conditions on the Newfoundland shelf during our study, we used an aggregated climate index previously calculated for this region; the index was the sum of many separate environmental time series, including North Atlantic Oscillation, ice cover and bottom and surface temperatures. All series were *z*-score transformed with means and standard deviations calculated based on 1981–2010 values, and scaled so that negative values in all series corresponded to colder conditions. The final aggregate index was calculated as the sum of all scaled series. The full method for calculating this series is in Colbourne *et al*. [48]. We calculated 5-year moving window averages of this index to smooth over short-term fluctuations, and to provide a better picture of the average climate fish would have experienced over their lifespans.

### 2.3. Spatial and spatio-temporal biomass distributions

As the distribution of trawl biomasses was heavy-tailed and zero-inflated, we used a two-stage robust estimator of average biomass in all of our analyses:

2.1 $$E_{\mathrm{zinf}-G}(b,m,n)\equiv \frac{m}{n}\;\exp\;\left(\Sigma_{i}\frac{\ln(b_{i})}{m}\right)\equiv \;\exp\;\left(E\left(\ln\left(b\cdot \frac{m}{n}\right)\right)\right),$$

where *n* is the number of observations in the sample, *m* the number of positive observations and *b_i_* are the positive values of biomass in each trawl for a given species *i*. This is equivalent to taking the geometric mean of all positive observations in a set, scaled by the fraction of all values in the set that are above zero. *E*_zinf-G_ was defined as equal to zero when there were no positive densities present. For all spatial analyses, averages were calculated for all trawls within a polygon within a year, otherwise the average was taken over all trawls from a given year. Standard errors for average biomasses within a year or polygon were calculated using a Jackknife estimator [49].

### 2.4. Community synchrony

Synchrony measures how closely fluctuations in the abundance of different species match one another over time or space. In this study, we used Loreau & de Mazancourt's [31] community synchrony estimator, calculated using the *synchrony* package for R [50]. This measure ranges between zero, when species dynamics are perfectly asynchronous, and one, when all species fluctuate up and down at the same time. We calculated community synchrony using a running 5-year window, ending in the year of interest. Statistical significance was determined using Monte Carlo randomizations of each time series, with 999 replicates.

### 2.5. Analysing changes in regional community composition

We used non-metric multidimensional scaling (NMDS) to illustrate shifts in community composition, standardizing it to changes in overall community biomass [51]. We calculated community dissimilarity between years as the Bray–Curtis dissimilarity based on the relative biomass of all species, using the *vegan* R package [52]. We then used NMDS to display this dissimilarity matrix in two dimensions, using the *metaMDS* function in the *vegan* package.

### 2.6. Analysing temporal changes in regional functional diversity

We quantified the community functional diversity as the biomass-weighted functional dispersion (FDis) of functional traits in the regional groundfish community on a yearly basis using the *FD* R package [53]. FDis provides a multivariate measure of the trait space of all species in the community, weighted by their relative abundances [37]. Whereas many measures of functional diversity are restricted to presence–absence data, FDis allowed us to quantify how changes in the relative abundance of the community resulted in changes in functional diversity. FDis also allowed us to combine quantitative and qualitative traits in our estimate of functional diversity. Functional diversity was weighted to the population biomass of the communities as this was the only index of abundance available to us. We calculated six functional traits comprised of numeric and categorical variables (electronic supplementary material, table S1): (i) *vertical position* refers to a categorical index of the depth distribution, (ii) *body length* is the maximum observed length (centimetres), (iii) *doubling time* is the average time it would take a population to double in size when starting from low density, (iv) *trophic level* is a numeric index assigning the estimated position in the food chain, (v) *aggregation* is a categorical index of the sociality or schooling behaviour, and (vi) *food niche* is a categorical classification based on the most prevalent prey items (M. Koen-Alonso, Fisheries and Oceans Canada 2014, personal communication). Traits i–iv were obtained from FishBase [54] using the *rfishbase* package in R [55]. *Aggregation* (trait v) was obtained through a primary literature search and communication with FishBase (electronic supplementary material, table S2). These functional traits were chosen because they reflect a broad range of the spatial, temporal and ecological aspects of each species, and reliable data were available for the full community of species.

### 2.7. Analysing spatio-temporal changes in community composition

To illustrate spatio-temporal changes in community composition, we grouped the trawls by polygon and by time period (1981–1984, 1985–1989, 1990–1994, 1995–2001, 2002–2006 and 2007–2013). These time periods were chosen such that most polygons had at least one trawl per polygon and period, and that no period overlapped during important dates (the start of the cod collapse in 1990 or the gear change in 1995).

The representative community type for each polygon in each period was determined through hierarchical complete linkage clustering [51], using the *hclust* function in base R. This clustering was based on the Bray–Curtis dissimilarity of the relative biomass between-polygon periods using the *vegan* package. We then partitioned the clustering tree into seven groups, chosen to give sufficient clusters to reveal large-scale changes in spatial structure while still being interpretable. While both aggregating communities into polygons and year groupings and the clustering procedure discard spatial information on community changes, they reduce variations in community composition due to sampling error and serve to give a useful and interpretable picture of how the community has changed across broad spatial and temporal scales.

To determine how the spatial structure of the groundfish community changed across time, we calculated how the amount of variance in community composition explained by geographical distance (*R*_distance_^2^) and difference in depth (*R*_depth_^2^) between surveys changed over time. We calculated the average density of each species for each polygon-year in the study period, standardized so that the density of each polygon-year summed to one. For all pairs of polygons in a year, we calculated the Bray–Curtis dissimilarity [51] between the communities, the geodesic distance between-polygon centroids, and the absolute difference in mean depths.

From each year, we estimated the regression of logit-transformed dissimilarity on log-transformed difference in depth and geographical distance and we calculated the partial adjusted *R*^2^ explained individually by depth and distance, using the *varpart* function from the *vegan* package. We chose logit and log transformations, as these best linearized the relationships between the predictors. The adjusted *R*^2^ values indicated how much of the variability in community composition in a given year could be explained due to linear change in divergence with space and depth between sites.

Prior to computing adjusted *R*^2^ values, we removed any polygon that did not have points in at least 30 of the 34 years of the study period, to ensure that any changes in the variance explained were not simply due to chances in spatial sampling extent. Eighty-two of the 150 polygons, representing the range of latitude and depths in the study area, met this criterion.

### 2.8. Measuring relative changes in community biomass and composition

We calculated time series of relative change over time for three community descriptors: cod biomass, total community biomass and community composition, measured by the Sørensen similarity index [51] of the standardized community in each year relative to the community in 1981. We scaled each time series by subtracting the minimum value observed, dividing by the value observed in 1981 and multiplying by 100. This resulted in three time series scaled between 0 (maximum distance from the value in 1981) and 100 (value in 1981).

## 3. Results

**Figure 1.**
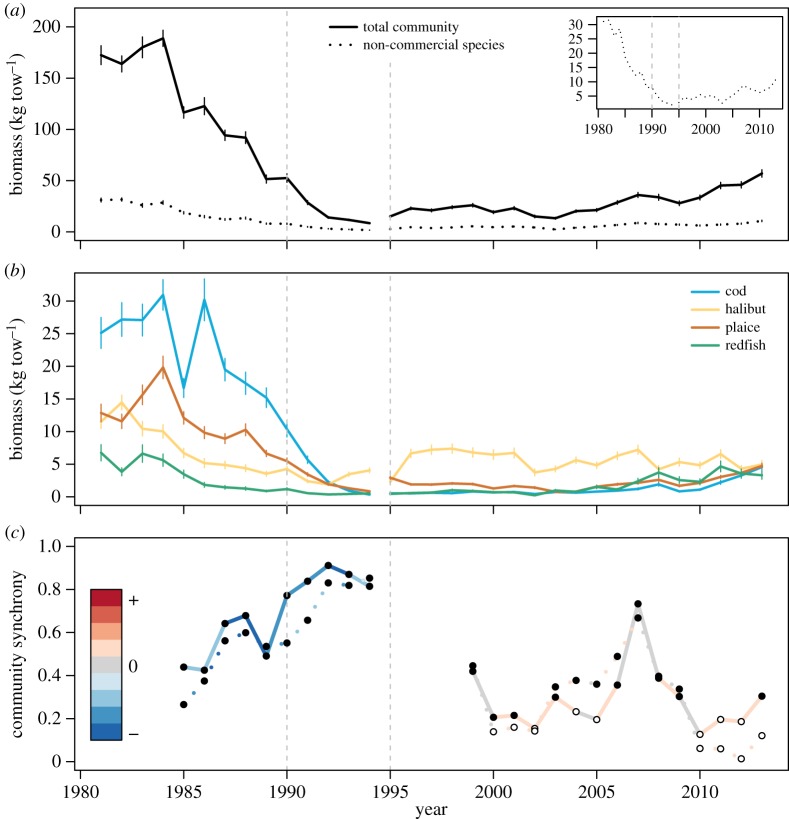
Trends in biomass and community synchrony. (*a*) Trends in mean population biomass of different community subsets: the total community (solid line) and the community excluding the commercially important species (dotted line). Vertical bars represent ±1 Jackknife standard errors (*n* = 421 (s.d. = 62) per year). The inset shows biomass dynamics of the non-commercial species alone, to show the scale of decline in this community. (*b*) Biomass trends for the four most abundant, commercially important species in the community. (*c*) Interspecific synchrony of community subsets. Full (empty) points denote statistically (in)significant community-wide synchrony in that community subset. Line colour denotes the direction of population change in that period: blue (red) denotes declining (increasing) biomasses in that community subset. Important transitions marked by vertical lines: 1990 is considered the start of the cod collapse, and the gear change occurred in 1995.

**Figure 2.**
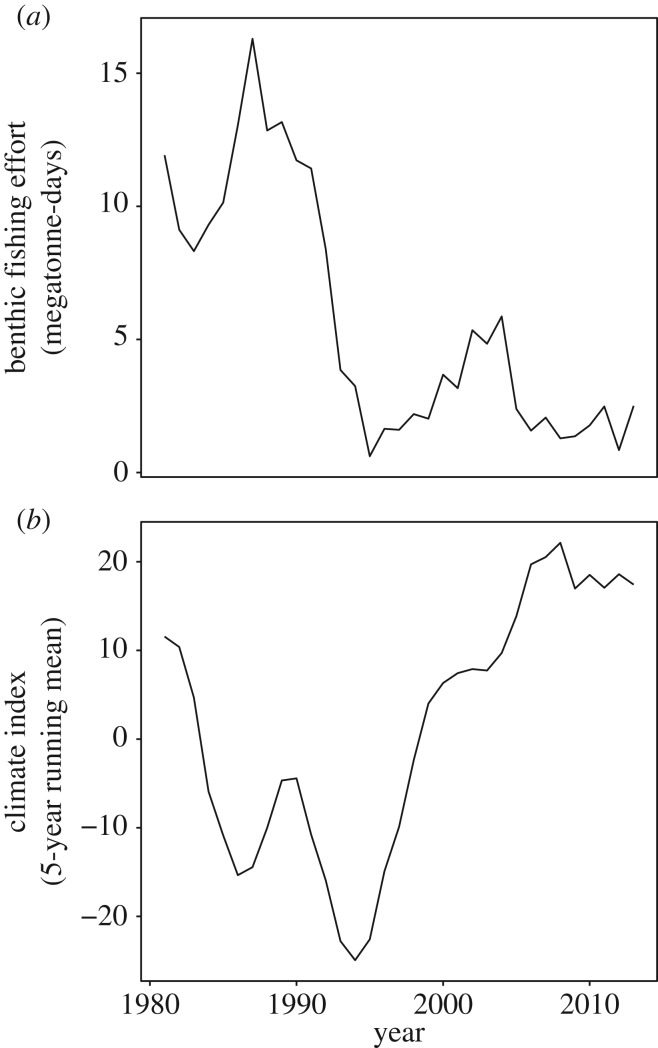
Trends in exogenous ecosystem drivers. (*a*) Time series of fishing effort focused on benthic fish, measured in megatonne-days. (*b*) Time series of the Newfoundland aggregated climatic index. The index is scaled so that negative values correspond to colder conditions.

Biomass collapse occurred from the mid-1980s to the early 1990s, with both the whole community (figure 1*a*, solid line) and the non-commercial fish community (figure 1*a*, dashed line) reaching a minimum in 1994. While the rapid decline of cod in the early 1990s was a major factor in the loss of community biomass (figure 1*b*, blue line), the other three commercial species showed biomass declines before the cod collapse (figure 1*b*, orange, yellow and green lines). Throughout the 1980s and early 1990s, leading up to and during the collapse, biomass fluctuations across the entire community became increasingly synchronous, with sustained biomass decreases resulting in the rapid loss of community biomass (figure 1*c*, solid line). These synchronous declines were not only driven by declines in commercial fish stocks, as the community excluding the primary commercially targeted species also showed the same patterns (figure 1*c*, dashed line). Community biomass remained low in the decade following the collapse, as species' biomass fluctuated independently (figure 1*a,c*). Modest increases in community biomass began in the early 2000s, initially driven by synchronous growth across the community (figure 1*a,c*). This recovery has continued since the mid-2000s but slowed or stalled during periods of asynchronous dynamics (e.g. halibut and cod) (figure 1*a,c*). The decline occurred during a period of high fishing effort, but the recovery did not coincide with the reduction in fishing effort (figure 2*a*). The overall climate (as measured by the aggregate climate index) was substantially colder than average (figure 2*b*), but both bottom temperatures and overall climate had largely returned to their prior state by 2000, again without substantial growth in fish stocks.

**Figure 3.**
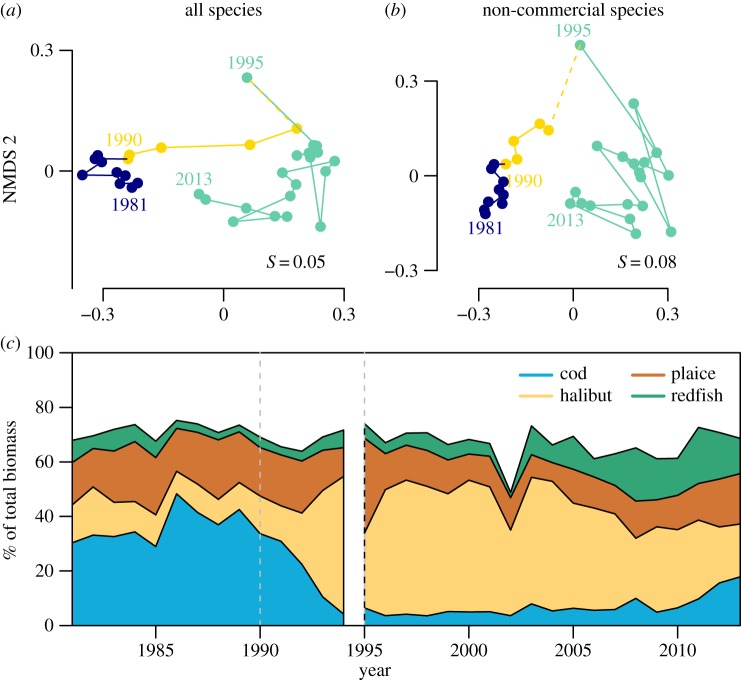
Changes in community composition across the study period. (*a,b*) Shifts in community composition illustrated by two-dimensional NMDS using the Bray–Curtis dissimilarity: (*a*) all species (*b*) non-commercial species. Colours denote key periods: blue are years prior to collapse, yellow are years following the collapse and light green are post-gear change years. (*c*) Shifts over time in the fraction of total community biomass held in the four most abundant species. Colours denote the four most abundant, commercially important species and white area denotes the remaining 26 non-commercial species. Vertical dashed lines are as in figure 1.

The collapse caused a major shift in community composition across the region (figure 3*a*, yellow; 1990–1994), which persisted over the next decade. Beginning in the mid-2000s, the community began to return towards its pre-collapse compositional state (figure 3*a*, green). This is most evident for the four most abundant species, where the collapse resulted in a shift in dominance from cod to halibut (figure 3*c*). Since the mid-2000s, the relative abundances of the four commercial species gradually shifted to a composition closer to that of the 1980s than at any time since. This trajectory is also present in the rest of the community (figure 3*b*), indicating that the compositional shift extends to the non-commercially targeted species. Recovery in these non-commercial species was slower, but estimates of their biomasses were more sensitive to the 1995 switch to smaller meshed Campelen trawl because of their smaller body sizes (electronic supplementary material, figure S5). Therefore, direct comparison of the pre- and post-1995 community is tenuous. The effects of this gear change are less evident for the large commercial species and the entire community (figure 3*a*), but present a problem for determining how far the current community is from its pre-collapse state.

**Figure 4.**
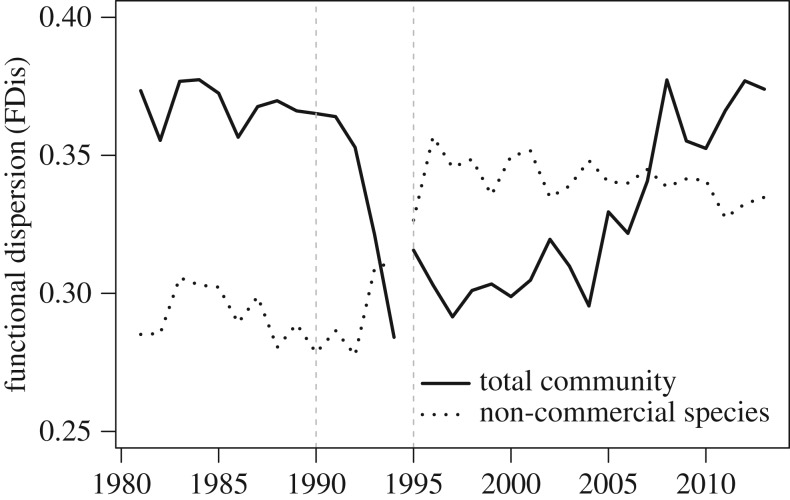
Time series of the biomass-weighted functional dispersion (FDis) of the regional groundfish community. FDis for all species (solid line) and the non-commercial species (dotted line) are shown. ±1 Jackknife standard errors (*n* = 421 (s.d. = 62) per year) are too small to be visible. Vertical lines are as in figure 1.

This trajectory of compositional change is reflected in the abundance-weighted functional dispersion of the community at the regional scale (figure 4). The major shift in composition in the early 1990s resulted in a loss of functional dispersion. This shift was the result of decreases in the functional dispersion of the commercial fish assemblage species, and was not compensated for by the increase in functional dispersion of the remaining species that occurred during this period. This loss of functional dispersion resulted from changes in the relative abundances of the functional traits for aggregation, doubling time and body length, whereas the traits for trophic level, food items and vertical position remained relatively constant (electronic supplementary material, figure S2). During the early 1990s, there was a proportional loss of schooling biomass, including cod, and a proportional increase in species with irregular aggregation, such as halibut and plaice (electronic supplementary material, figure S2*a*). The biomass-weighted mean doubling time increased (electronic supplementary material, figure S2*d*) and the biomass-weighted mean body length decreased (electronic supplementary material, figure S2*f*). Functional dispersion remained low until the mid-2000s, but has since increased, returning to pre-collapse levels (figure 4). The increase in functional dispersion corresponded with increasing mean schooling biomass, increases in mean body length and reductions in mean doubling time, although none of the average values of these traits have fully returned to their pre-collapse levels (electronic supplementary material, figure S2).

**Figure 5.**
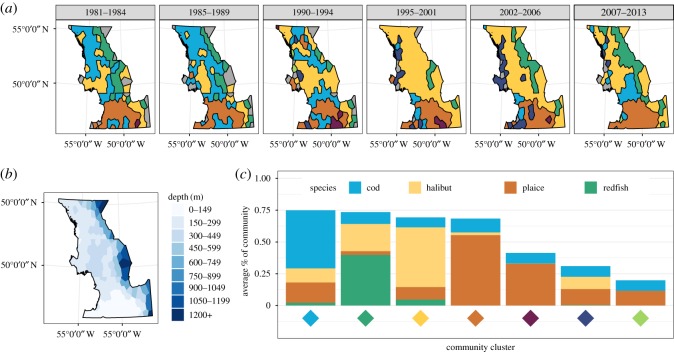
Change in spatial arrangement of the groundfish community over time. (*a*) Changes in spatial distribution of community clusters. Grey polygons did not have surveys in that period. (*b*) Mean depth of each polygon. (*c*) Mean fractions of the top four species in each of the seven clusters in (*a*). Area of each bar represents the fraction of that species averaged across all polygon-years for that cluster.

The collapse was characterized by declining abundances of cod throughout the region (electronic supplementary material, figure S3) and by the spatial homogenization of community composition (figure 5). In the early 1980s, regional composition was spatially structured, with halibut and redfish dominating in deeper waters, cod dominating in mid-depths and plaice dominating in shallower southern waters (figure 5). Between 1995 and 2006, cod-dominated communities were all but absent, replaced by the expansion of halibut-dominated communities and those in which the four large commercial groundfish species comprised a subordinate component of the biomass. Since 2007, cod biomass has increased and regained dominance in central shelf waters. However, this has not occurred in other parts of the region, such as the north, where cod was most dominant before the collapse. Regional increases in the biomass of redfish throughout the 2000s resulted in a similar return of its dominance along the edge of shelf (figure 5).

**Figure 6.**
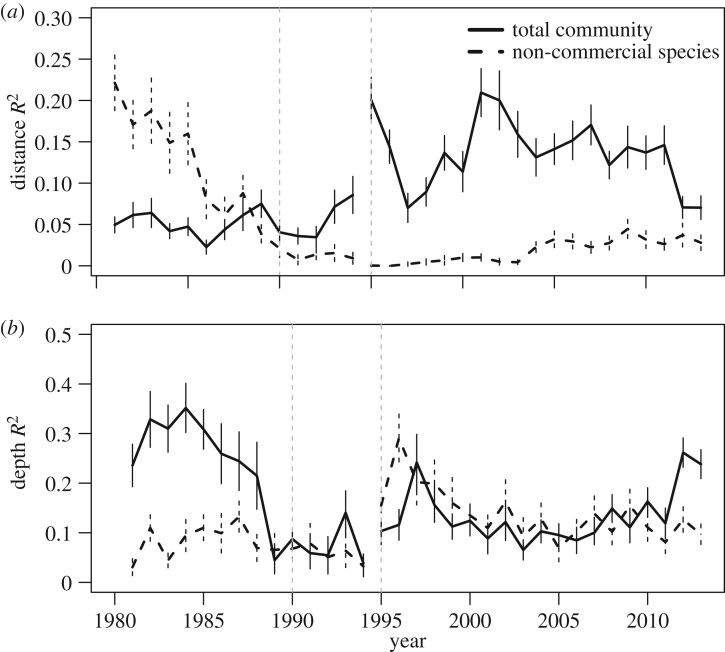
Changes over time in variance of community composition explained by (*a*) geographical distance and (*b*) differences in depth between sites. Lines indicate estimated value, with vertical bars indicating ±1 Jackknife standard error (*n* = 421 (s.d. = 62) per year). Separate trends are plotted for all species (solid line) and the non-commercial species (dashed line).

We observed two large-scale shifts in spatial and depth structure of the overall community (figure 6): the community became less depth stratified (figure 6*b*) immediately prior to the collapse and more strongly spatially stratified following the collapse (figure 6*a*). These trends have both partially reversed from 2010 onwards. The non-commercial species (figure 6 dashed line) showed the opposite trend: this community was strongly spatially stratified (figure 6*a*), and showed no depth stratification (figure 6*b*) before the collapse. Prior to the cod collapse, the spatial structure of the non-commercial species declined sharply and has not increased subsequently. Because we were measuring spatial and depth structure, the declines in observed spatial structure could have been the result of an increase in total community variance, even if the community spatial pattern remained constant. However, we did not observe an increase in community variance; average *alpha* diversity declined during the collapse (electronic supplementary material, figure S4*a*) and while both average between-polygon dissimilarity (electronic supplementary material, figure S4*b*) and the variance of dissimilarity (electronic supplementary material, figure S4*c*) increased, this only occurred following the collapse in biomass.

**Figure 7.**
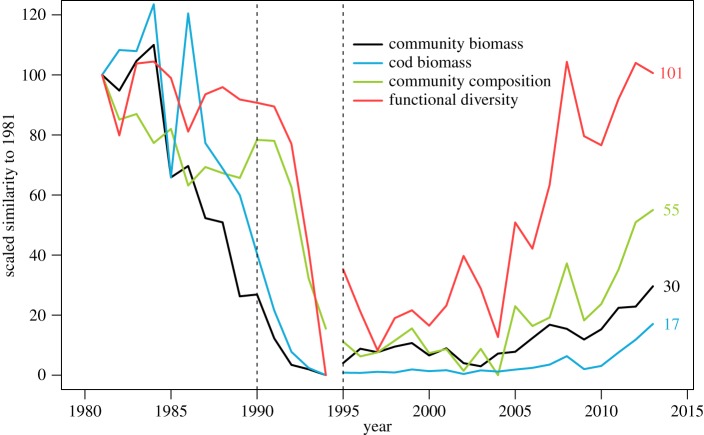
Relative change of different community metrics over time. Each line has been scaled between 0 and 100, where 100 is equal to the reference value in 1981. Black: mean per-trawl biomass; light blue: mean per-trawl cod biomass; green: community composition, measured as the Bray–Curtis dissimilarity of the community in a given year from the community in 1981; red: functional dispersion. Vertical lines are as in figure 1.

We documented variability in the rate of change in biomass, composition and functional diversity over time during the collapse and recovery. The shift in community composition occurred after the biomass collapse, and recovery to pre-collapse composition occurred faster than the recovery of biomass itself (figure 7). The biomass collapse of cod and the groundfish community began in the mid to late 1980s. By contrast, the community composition remained relatively constant until 1990. Since 2009, cod biomass has increased, but this only represents a 17% recovery of the biomass that was lost. By contrast, the community as a whole is showing stronger signs of recovery, regaining 31% of its biomass and 55% of its composition since 1994, measured as the Bray–Curtis dissimilarity from the 1981 reference composition. Functional dispersion showed the strongest recovery, and by 2013 had returned to its pre-collapse reference level. While figure 7 only showed trends for the whole community (including the commercially fished species), the same recovery trends held for the non-commercial stocks considered in isolation (not shown).

The timing of the collapse of biomass (figure 7) corresponded with periods of declining bottom temperatures and high fishing pressure, from 1981 until 1992 (figure 2). However, the rapid changes observed in community composition and functional diversity occurred after temperatures had begun increasing and fishing pressure was sharply reduced, from 1992 onward. Further, neither the rate of biomass nor community composition recovery were closely associated with changes in environmental conditions.

## 4. Discussion

The dynamics of the Northwest Atlantic cod are embedded within those of the broader groundfish community. The collapse of the cod in the early 1990s was preceded by increasingly synchronous and negative biomass fluctuations in commercially targeted and non-targeted species alike. The collapse caused a compositional reorganization of the community, and a loss of its spatial structure and functional diversity. Now, more than two decades after the establishment of the moratorium, the groundfish community, as a whole, is showing strong signs of recovery. Overall, rather than swift and spatially homogeneous, the recovery of groundfish populations has been slow and spatially heterogeneous, varying significantly across the region and from year to year.

The increase in community synchrony leading up to and during the biomass collapse shows that the changes in the ecosystem were impacting the entire groundfish community. The increasingly synchronous declines in biomass were not limited to the commercially targeted species, suggesting that the entire community was responding to common drivers. These drivers probably include the unusually cold surface waters reported during that period [11] (figure 2*b*) targeting a functionally less diverse groundfish community, and high community-wide mortality from indiscriminate bottom-trawl fishing (figure 2*a*). Whereas there are almost no records of by-catch from fishing from the period of the decline, by-catch could have been a large source of mortality for many species with limited commercial value (although see [56]). Overfishing probably reduced the resilience of this community [57], making it more susceptible to other drivers contributing to the collapse, such as fisheries by-catch, environmental change and reduced biomass of forage fish [11,17,58]. Further investigation is needed to understand why the collapse was so pervasive across the community. This is in contrast with the nearby Scotian shelf, where the collapse of commercially targeted predator biomass led to an increase in forage fish [19].

The compositional rearrangement occurred following the biomass collapse, but the relative community composition is now showing signs that it is in the process of reverting back to its pre-collapsed state. This community reorganization occurred because of changes in the relative abundances of all species, but it was especially apparent in the shift in regional dominance from cod to halibut. The compositional rearrangement is consistent with the theory that the cod recovery was slowed through reduced forage fish abundance [22], and by competition and juvenile predation from species that became dominant post-collapse [21]. While invertebrate abundances (primarily crab and shrimp) increased substantially following the groundfish collapse [19,59] and could have provided an alternative food source for cod and other predatory fish [60], these species do not have the same nutritional value for groundfish and may not have provided a sufficient food source to rebuild stock abundances [61]. The recovery of groundfish stocks may also be contributing to the observed declines in invertebrate stocks through increased predation rates. As there is almost no data on pre-1995 invertebrate densities, it is difficult to tell whether these declines represent a return to a pre-existing state or represent a substantial departure from historical norms.

The current trajectory of the community towards its pre-collapse composition is a promising sign of recovery. However, it is uncertain whether the Newfoundland shelf ecosystem will ever fully return to its previous state. Warming temperatures, species interactions and selection for smaller bodied individuals driven by centuries of fishing pressure may direct the community towards a new dynamical regime [15,35,62,63]. The nearby Grand Banks groundfish community has also shown a similar pattern of rapid compositional rearrangement and return from 1990 until present [24], but as that study did not extend to the time period prior to the collapse it is difficult to judge the extent to which that community has returned to its prior state. The dynamics of the Newfoundland shelf groundfish community appear to differ from those on the Scotian shelf [17,21]; while predator biomass declined and invertebrate densities increased in both systems, capelin, the primary forage fish in both systems, declined in the Newfoundland shelf but increased off the Scotian shelf. The loss of top predators in the 1990s did cause compositional rearrangement in both ecosystems [19], and there are indications that both may now be in the process of reverting back to their previous states [21]. We also observed a similar increase in *beta* diversity (as measured here by average community dissimilarity) that was reported following the collapse of cod on the Scotian shelf [30], although we did not observe the same increase in *alpha* diversity that occurred there. This may imply that cod were also acting as a homogenizing predator in the Newfoundland shelf by reducing spatial inequalities prior to its collapse.

The compositional rearrangement following the biomass collapse resulted in a loss of functional diversity. This loss occurred because the community became dominated by smaller species with longer population doubling times and reflects a community prone to more rapid and synchronous response to external stressors. A similar loss of functional diversity was hypothesized to have occurred on the Scotian shelf where the collapse was associated with a shift to smaller bodied species [18]. That the functional dispersion in the Newfoundland community has made a full recovery to pre-collapse levels is notable, especially because the composition has yet to make a full recovery. None of the three average trait values affected by the compositional shift have yet returned to their pre-collapse means, which indicates that, while the overall functional diversity of the community is similar to what it was before the collapse, the functional composition still remains altered. Still, functional diversity tends to be correlated with ecosystem processes such as productivity [64,65]. This result indicates that functional diversity may be an important feature of the recovery in the functioning of the groundfish community.

The collapse of the community affected the entire sampled region, yet, to date, the recovery is most evident in central mid-depth waters. The collapse and reorganization of the community caused the spatial homogenization of the region, as cod- and redfish-dominated communities throughout the region were lost, most often replaced by those dominated by halibut. This is consistent with the collapse of cod representing an alternative community state [18,21], as large-scale spatial homogenization is expected to occur prior to or during ecological regime shifts [39]. Further, the loss of long-distance spatial structure, as indicated by the decline in community dissimilarity, is similar to what occurred on the Eastern Scotian shelf during the collapse of that groundfish community, and may indicate that spatial connectivity stabilized the collapsed community state, slowing recovery [38].

In contrast with the regional signature of the collapse, recent signs of recovery (2007–2013), especially for cod, have been local and aggregated. The localized nature of this recovery warrants further investigation. Recovery may be driven by spatially varying patterns of population productivity and natural or fishing mortality [20,35]. Alternatively, predation of juveniles by mid-sized fish and macroinvertebrates may have impeded the recovery of cod and other slow-growing fish [66], and the local recovery represents the first signs that cod are overcoming those depensatory mechanisms. If recovery is primarily limited by external factors, the spatial community structure should globally recover as the community recovers [38,67]. If recovery is limited by depensatory effects, the recovery should occur in smaller regions, and spread more slowly than the rate of dispersal [41,42], as new regions will only be able to recover when sufficient recruits from neighbouring areas can arrive to overcome the depensatory effects. This highlights the usefulness of monitoring spatial community dynamics as a signature of recovery, as it can allow us to distinguish between alternative ecosystem drivers and determine appropriate management responses.

The community signatures presented in our study reveal changes occurring at different time scales [43,68] and it is evident that the shift in community composition followed the biomass collapse, but that compositional and functional recovery are occurring faster than biomass or cod recovery. The biomass collapse of cod and the groundfish community began in the mid to late 1980s. By contrast, the community composition remained relatively constant until 1990. This delay indicates that the community completely reorganized as a result of the loss of biomass, particularly for the previously dominant top predator, cod. Since 2009, cod biomass has increased, but this only represents a 17% recovery of the biomass that was lost. Although the community in 1981 had been under fishing pressure for centuries and therefore does not represent a pristine system [12], this year represents our best estimate of the community prior to its collapse. By contrast, the community as a whole is showing stronger signs of recovery, regaining 31% of its biomass and 55% of its composition since 1994, measured as the Bray–Curtis distance from the 1981 reference composition. The greatest recovery has been in the regional functional diversity, which has completely returned to its pre-collapse level. This compositional recovery may facilitate the further recovery of cod biomass, especially if its recovery has been limited by the lack of forage fish [22], or by competition and predation from species dominant after the collapse [15]. Given the gear change in 1995, our results probably overestimate the magnitude of cod and total biomass recovery (see electronic supplementary material, figure S6*a*), as it entailed a switch to a smaller meshed net, which is expected to catch more biomass per area trawled. However, this is not expected to bias our estimates of the recovery in species composition, as this metric is based on relative abundances (electronic supplementary material, figure S6*b–e*). As such, it is possible that the community composition and functional diversity have actually recovered more rapidly relative to biomass than these analyses suggest. Taken together, these findings show that this ecosystem remains far from its pre-collapse state, but that recovery is ongoing [69].

One of the major research topics on regime shifts is early warning signs: indicators such as temporal or spatial variance or autocorrelation which increase prior to the regime shift itself [70]. While we did not test for increasing variance prior to the collapse in this system, as there were too few years of pre-collapse data to accurately calculate variances or detect variance trends, we believe that community-based indicators may be useful for detecting future fisheries regime shifts. In complex communities, the species most prone to collapse may not show any early warning signals which may appear in the dynamics of other species in the system [71]. Using multi-species equivalents of variance such as community synchrony, or equivalents of spatial autocorrelation such as spatial community dissimilarity may increase the power of these community-based early warning signs.

Our findings demonstrate how shifting the focus of analysis from one or a few commercial species to the spatial ecology of the entire community [7,24] reveals new signatures of the collapse and recovery of an ecosystem. Synchronous shifts in abundance among populations and changes in the spatial community structure can highlight the onset of regime shifts, before these shifts become apparent as biomass declines. Similarly, shifts in functional diversity towards pre-collapse state can be early signs of onset in recovery. These community signatures integrate across a broad range of taxa and track departures from target or baseline conditions. Future research should focus on deepening the analysis of community change in response to factors such as climate, fishing pressure and other aspects of the marine food web, beyond the groundfish community.

Our approach can be extended to understand collapses in other ecosystems. Indeed, monitoring community dynamics can serve to prompt earlier implementation of ecosystem-based management strategies than can build on single-species stock assessments by providing an integrated perspective of changes occurring in multiple stocks [6]. To identify potential risks to marine resources, fisheries managers have identified the need for more information on the state of the marine environment and trophic interactions, including robust indicators of when regime shifts have occurred and better tools for tracking the spatial distribution of species assemblages over time [72]. Our study shows how combining community, functional and spatial indicators can give new insights into regime changes in marine communities, and can inform long-term management of the ecosystems in which these communities are embedded.

## Supplementary Material

Supplementary Materials

## Supplementary Material

Groundfish data for analysis

## Data accessibility

The code and data used for analyses is available at http://dx.doi.org/10.5281/zenodo.818096.
