# Supplementary material for: Signatures of the collapse and incipient recovery of an overexploited marine ecosystem
Source: R Soc Open Sci. 2017 Jul 5;4(7):170215. doi: 10.1098/rsos.170215 (PMC5541544; doi:10.1098/rsos.170215)
Supplement: Supplementary Materials [file rsos170215supp1.docx]

Supplementary Materials:

**Table S1.** List of functional traits and associated levels or ranges for the species included in the functional analysis. All traits are in reference to the adult life history.

| **Trait** | **Description** | **Levels** | **Source** |
| --- | --- | --- | --- |
| Vertical position | Categorical index depth distribution | demersal, benthopelagic, bathypelagic, bathydemersal, pelagic | Fishbase.org |
| Length  (cm) | Maximum observed length | 7.6-200 (cm) | Fishbase.org |
| Doubling time  (Years) | Average time for a strongly reduced population to double in size after the cessation of fishing | 1.25-14 (years) | Fishbase.org |
| Trophic Level | Numerical index of food-web position | 3.0-4.5 | Fishbase.org |
| Aggregation | Categorical index of sociality or schooling behaviour | none = no indication of particular aggregations of individuals  rare = rare observation of schooling or shoaling behaviour  irregular = sometimes schooling or shoaling; often only in 1 lifestage (adult or juvenile); brooders; migration  shoal = regular shoaling; i.e., aggregation of individuals of a population with social interactions  schools = regular schooling behavior (not only for spawning); i.e., directed aggregated movement | Fishbase.org  Personal comm.  Literature search |
| Food niche | Categorical classification of adult prey items | planktivore, plankpiscivore, piscivore, small benthivore, medium benthivore, large benthivore | DFO Canada |

**Table S2.** List of species retained in analysis and according functional traits. Sources are given where trait information was not retrieved directly through fishbase.org.

| **Species** | **Vertical Position** | **Length (cm)** | **Doubling Time (yr)*** | **Trophic Level*** | **Aggregation** | **Food niche*** |
| --- | --- | --- | --- | --- | --- | --- |
| *Amblyraja radiata* | demersal [1] | 105 [2] | 9.25 | 4 | rare^a^ | Large Benthivore |
| *Anarhichas denticulatus* | benthopelagic [1] | 180 [3] | 9.25 | 3.8 | rare^a^ | Large Benthivore |
| *Anarhichas lupus* | demersal [1] | 150 [3] | 9.25 | 3.2 | irregular^a^ | Large Benthivore |
| *Anarhichas minor* | demersal [1] | 180 [3] | 9.25 | 3.5 | irregular^a^ | Large Benthivore |
| *Bathyraja spinicauda* | bathydemersal [4] | 170 [5] | 9.25 | 4 | irregular^a^ | Large Benthivore |
| *Centroscyllium fabricii* | bathydemersal | 107 [6] | 9.25 | 3.9 | irregular^a^ | Piscivore |
| *Coryphaenoides rupestris* | bathypelagic [4] | 110 [7] | 9.25 | 3.5 | schools^a^ | Medium Benthivore |
| *Cottunculus microps* | bathydemersal [4] | 30 [7] | 9.25 | 3.4 | none^b^ | Small Benthivore |
| *Cyclopterus lumpus* | benthopelagic [1] | 61 [4] | 9.25 | 3.9 | irregular^a^ | Medium Benthivore |
| *Enchelyopus cimbrius* | demersal [1] | 41 [8] | 2.9 | 3.5 | rare^a^ | Small Benthivore |
| *Eumicrotremus spinosus* | demersal [9] | 13.2 [4] | 2.9 | 3.5 | none [10] | Small Benthivore |
| *Gadus morhua* | benthopelagic [1] | 200 [8] | 2.9 | 4.4 | schools^a^ [8] | Piscivore |
| *Glyptocephalus cynoglossus* | demersal [1] | 60 [11] | 9.25 | 3.1 | irregular^a^ | Medium Benthivore |
| *Hippoglossoides platessoides* | demersal [1] | 82.6 [4] | 9.25 | 3.7 | irregular^a^ | Large Benthivore |
| *Limanda ferruginea* | demersal [12] | 64 [3] | 2.9 | 3.2 | irregular^a^ | Medium Benthivore |
| *Lycodes esmarkii* | bathydemersal [4] | 75 [13] | 9.25 | 3.4 | rare^a,c^ | Medium Benthivore |
| *Lycodes reticulatus* | bathydemersal [4] | 36 [3] | 2.9 | 3.5 | rare^a,c^ | Medium Benthivore |
| *Lycodes vahlii* | bathydemersal [4] | 52 [13] | 9.25 | 3.4 | rare^a,c^ | Medium Benthivore |
| *Macrourus berglax* | benthopelagic [8] | 110 [7] | 14 | 4.5 | rare^a,c^ | Large Benthivore |
| *Malacoraja senta* | bathydemersal [1] | 61 [14] | 9.25 | 3.5 | irregular^a^ | Medium Benthivore |
| *Myoxocephalus octodecemspinosus* | demersal [15] | 46 [3] | 1.25 | 3.5 | shoal^a^ | Medium Benthivore |
| *Myoxocephalus scorpius* | demersal [9] | 60 [16] | 2.9 | 3.9 | shoal^a^ | Medium Benthivore |
|  |  |  |  |  |  |  |
| **Species** | **Vertical Position** | **Length (cm)** | **Doubling Time (yr)*** | **Trophic Level*** | **Aggregation** | **Food niche*** |
| *Nezumia bairdii* | benthopelagic | 40 [17] | 2.9 | 3.6 | none^b^ | Small Benthivore |
| *Notacanthus chemnitzii* | benthopelagic [4] | 120 [18] | 9.25 | 3.5 | none^a^ | Large Benthivore |
| *Notacanthidae* | benthopelagic [4] | 120 [18] | 9.25 | 3.5 | none^a^ | Large Benthivore |
| *Phycis chesteri* | benthopelagic [19] | 42 [4] | 9.25 | 3.2 | none [20] | PlankPiscivore |
| *Reinhardtius hippoglossoides* | benthopelagic [1] | 80 [21] | 9.25 | 4.5 | irregular^a^ | Piscivore |
| *Sebastes mentella* | bathypelagic [1] | 58 [4] | 14 | 3.7 | shoal^a^ | PlankPiscivore |
| *Sebastes norvegicus* | pelagic-oceanic [1] | 100 [22] | 9.25 | 4 | shoal^c^ | PlankPiscivore |
| *Urophycis tenuis* | demersal [1] | 133 [23] | 9.25 | 4.2 | shoal^a^ | Piscivore |

* Doubling time and trophic level are based off of calculations using demographic and feeding information in Fishbase [24]. Food niche is based on pers. comm., Mariano Koen (DFO Canada, 2014).

a = These values were based on information and sources about 'Association' from pers. Communication from Maria Lourdes Palomares, Fishbase, 2014.

b = assumed from catch data in this study; any fish that was only every caught singly in a net tow was assumed to not aggregate.

c = pers. observation by Pierre Pepin (DFO Canada, 2014).

## References for trait data

1. Riede K. 2004 Global register of migratory species: from global to regional scales: final report of the R&D-Projekt.

2. Sulikowski J, Kneebone J, Elzey S, Jurek J, Danley P, Howell W, Tsang P. 2005 Age and growth estimates of the thorny skate (*Amblyraja radiata*) in the western Gulf of Maine. *Fish. Bull.*

3. Robins CR, Ray GC, Douglass J, Freund R. 1986 A field guide to Atlantic coast fishes of North America. *Peterson Field Guide Ser. Houghton Miffiin Boston*

4. Coad BW, Reist JD. 2004 Annotated list of the Arctic marine fishes of Canada. , iv:+112 p.

5. Stehmann M, Bürkel DL. 1986 Rajidae. In *Fishes of the north-eastern Atlantic and the Mediterranean* (eds PJP Whitehead, M-L Bauchot, J-C Hureau, J Nielsen, E Tortonese), pp. 163–196. UNESCO.

6. Compagno L. 1984 FAO species catalogue: vol 4 sharks of the world: an annotated and illustrated catalogue of shark species known to date. *FAO Fish. Synop.* , 1–249.

7. Muus BJ, Nielsen JG. 1999 *Sea fish*. Scandinavian Fishing Year Book.

8. Cohen DMI, Iwamoto T, Scialabba T, Whitehead N, Palmer PJ, Cohen DM. 1990 FAO species catalogue: vol. 10 gadiform fishes of the world (order gadiformes), an annotated and ilustrated catalogue of Cods. Hakes, grenadiers and other gadiform fishes known to date.

9. Parin NV, Fedorov VV, Sheiko BA. 2002 An annotated catalogue of fish-like vertebrates and fishes of the seas of Russia and adjacent countries: Part 2. Order Scorpaeniformes. *J. Ichthyol.* **42**, S60–S135.

10. Whitehead PJP, Bauchot ML, Hureau JC, Nielsen J, Tortonese E, editors. 1986 *Fishes of the North-eastern Atlantic and the Mediterranean*. Paris: UNESCO.

11. Nielsen JG. 1986 Pleuronectidae. In *Fishes of the north-eastern Atlantic and the Mediterranean* (eds PJP Whitehead, M-L Bauchot, J-C Hureau, J Nielsen, E Tortonese), pp. 1299–1307. UNESCO.

12. Scott WB, Scott MG. 1988 Atlantic fishes of Canada. *Can. Bull. Fish. Aquat. Sci.* **219**.

13. Andriashev AP. 1986 Zoarcidae. In *Fishes of the north-eastern Atlantic and the Mediterranean* (eds PJP Whitehead, M-L Bauchot, J-C Hureau, J Nielsen, E Tortonese), pp. 1211–1229. UNESCO.

14. Bigelow HB, Schroeder WC. 1953 Sawfishes, guitarfishes, skates and rays. In *Fishes of the western North Atlantic. Part two* (eds J Tee-Van, et. al), New Haven: Sears Foundation for Marine Research, Yale University Press.

15. Murdy EO, Birdsong RS, Musick JA. 1997 *Fishes of Chesapeake Bay*. Washington: Smithsonian Institution Press. See http://agris.fao.org/agris-search/search.do?recordID=US201300038427.

16. Nielsen JG, Bertelsen E. 1992 Fisk i grønlandske farvande.

17. Cohen DMI, Iwamoto T, Scialabba T, Whitehead N, Palmer PJ, Cohen DM. 1990 FAO species catalogue: vol. 10 gadiform fishes of the world (order gadiformes), an annotated and ilustrated catalogue of Cods. Hakes, grenadiers and other gadiform fishes known to date.

18. Sulak KJ. 1990 Notacanthidae. In *Check-list of the fishes of the eastern tropical Atlantic* (eds JC Quero, JC Hureau, C Karrer, A Post), UNESCO.

19. Haedrich RL, Merrett NR. 1988 Summary atlas of deep-living demersal fishes in the North Atlantic Basin. *J. Nat. Hist.* **22**, 1325–1362.

20. Methven DA, McKelvie DS. 1986 Distribution of Phycis chesteri (Pisces: Gadidae) on the Grand Bank and Labrador Shelf. *Copeia* **1986**, 886–891. (doi:10.2307/1445284)

21. Orlov A, Binohlan C. 2009 Length–weight relationships of deep-sea fishes from the western Bering Sea. *J. Appl. Ichthyol.* **25**, 223–227.

22. Hureau J-C, Litvinenko NI. 1986 Scorpaenidae. In *Fishes of the north-eastern Atlantic and the Mediterranean* (eds PJP Whitehead, M-L Bauchot, J-C Hureau, J Nielsen, E Tortonese), pp. 1211–1229. UNESCO.

23. IGFA. 2001 Database of IGFA angling records until 2001. *IGFA Ft. Lauderdale USA*

24. Froese R, Pauly D. 2016 Fishbase. See www.fishbase.org.

**
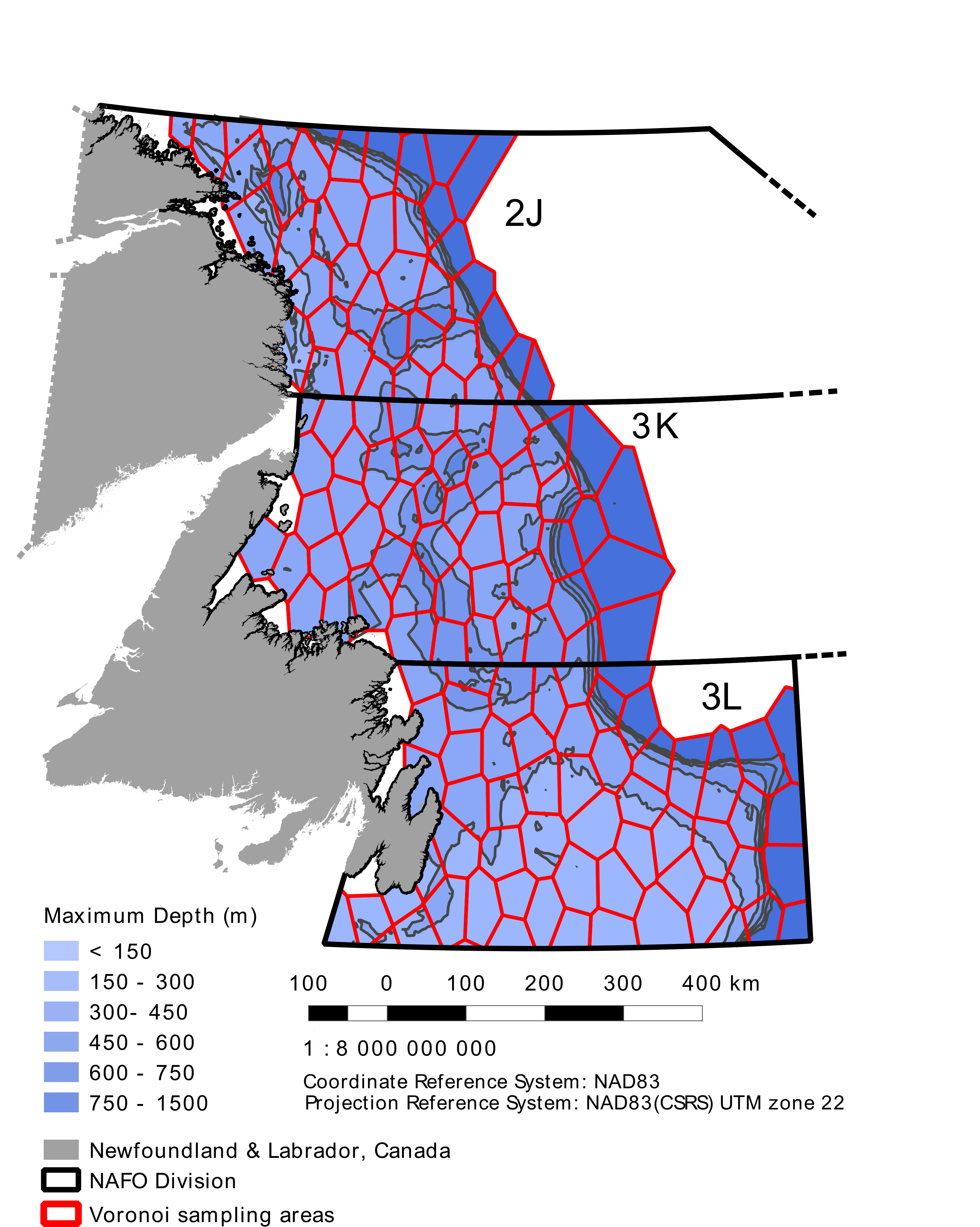
**

**Figure S1.** DFO survey study area. The polygon grid used for spatial analyses is shown in red. Blue shading indicates the average depth of that polygon, estimated from trawl depths.

**
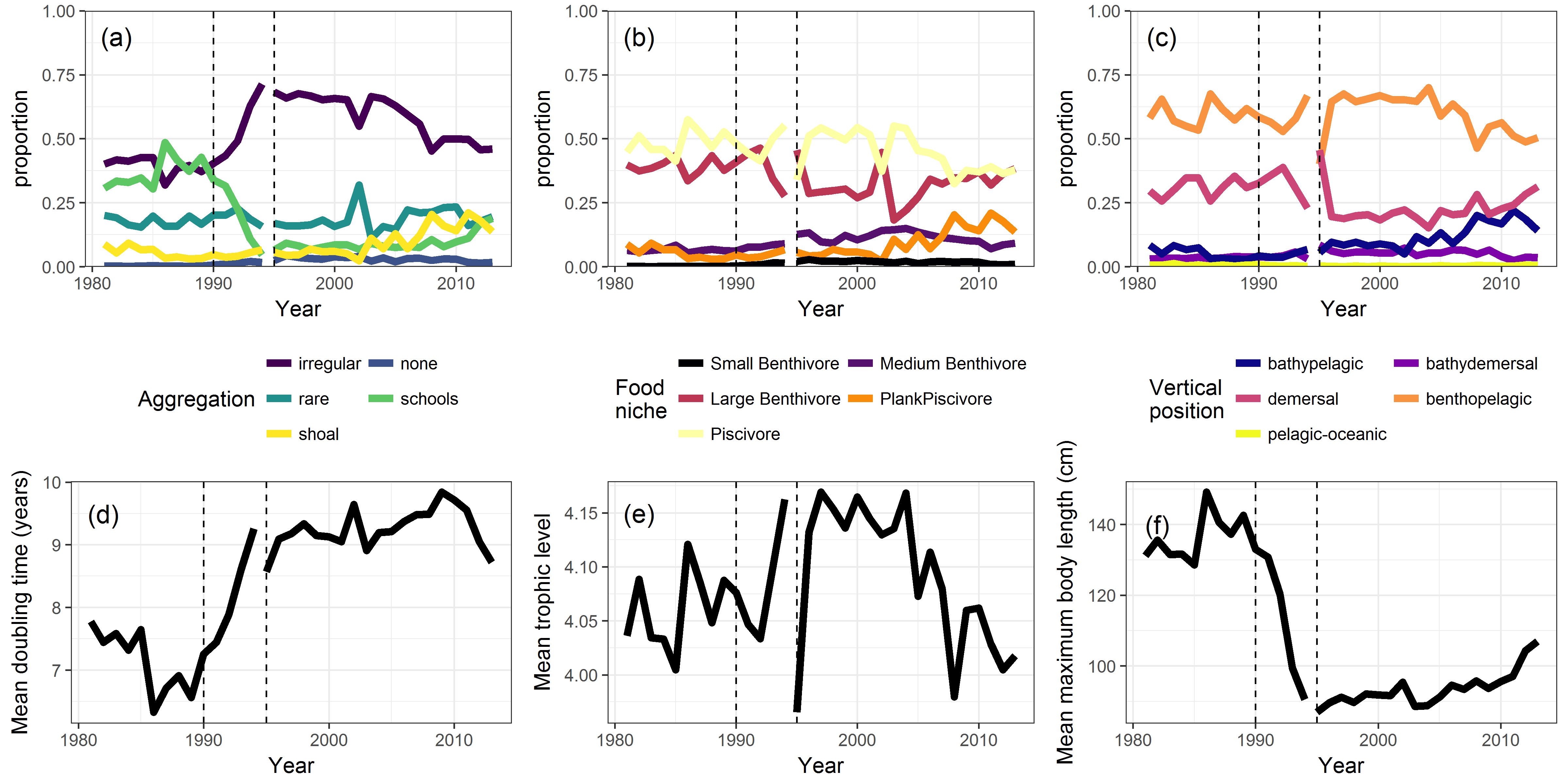
**

**Figure S2.** Changes in biomass-weighted mean trait values across the study area. (a) Relative biomass of each aggregation category in each year of the dataset. (b) Relative biomass of each food niche category in each year of the dataset. (c) Relative biomass of each vertical position category in each year of the dataset. (d) Biomass weighted mean doubling time (years) in each year of the dataset. (e) Biomass weighted mean trophic level in each year of the dataset. (f) Biomass weighted mean body length (cm) in each year of the dataset.


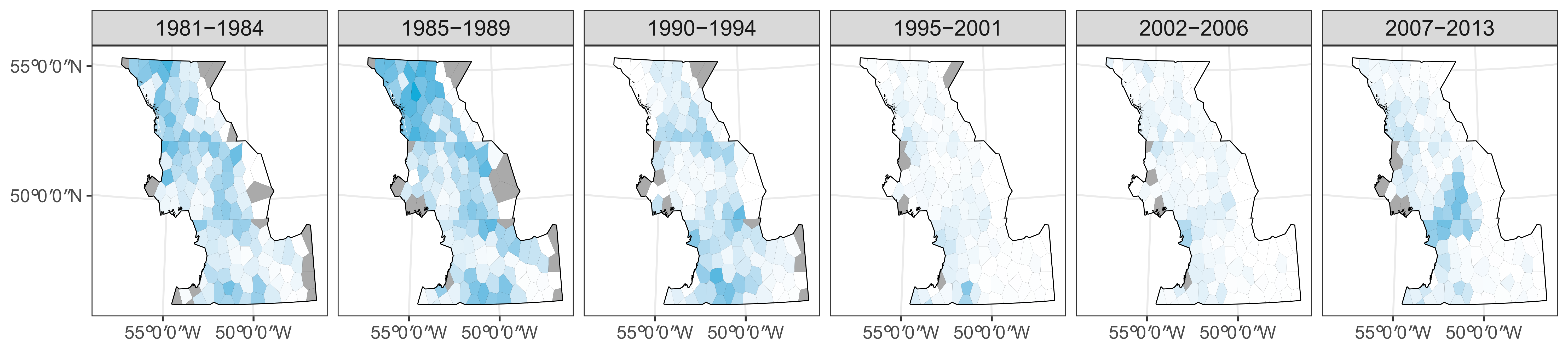


**Figure S3.** Changes in the spatial distribution of mean per-trawl abundance of cod. White indicates no cod present and fully saturated blue indicates the highest cod abundance observed during the study period. Grey polygons were not surveyed in that period.


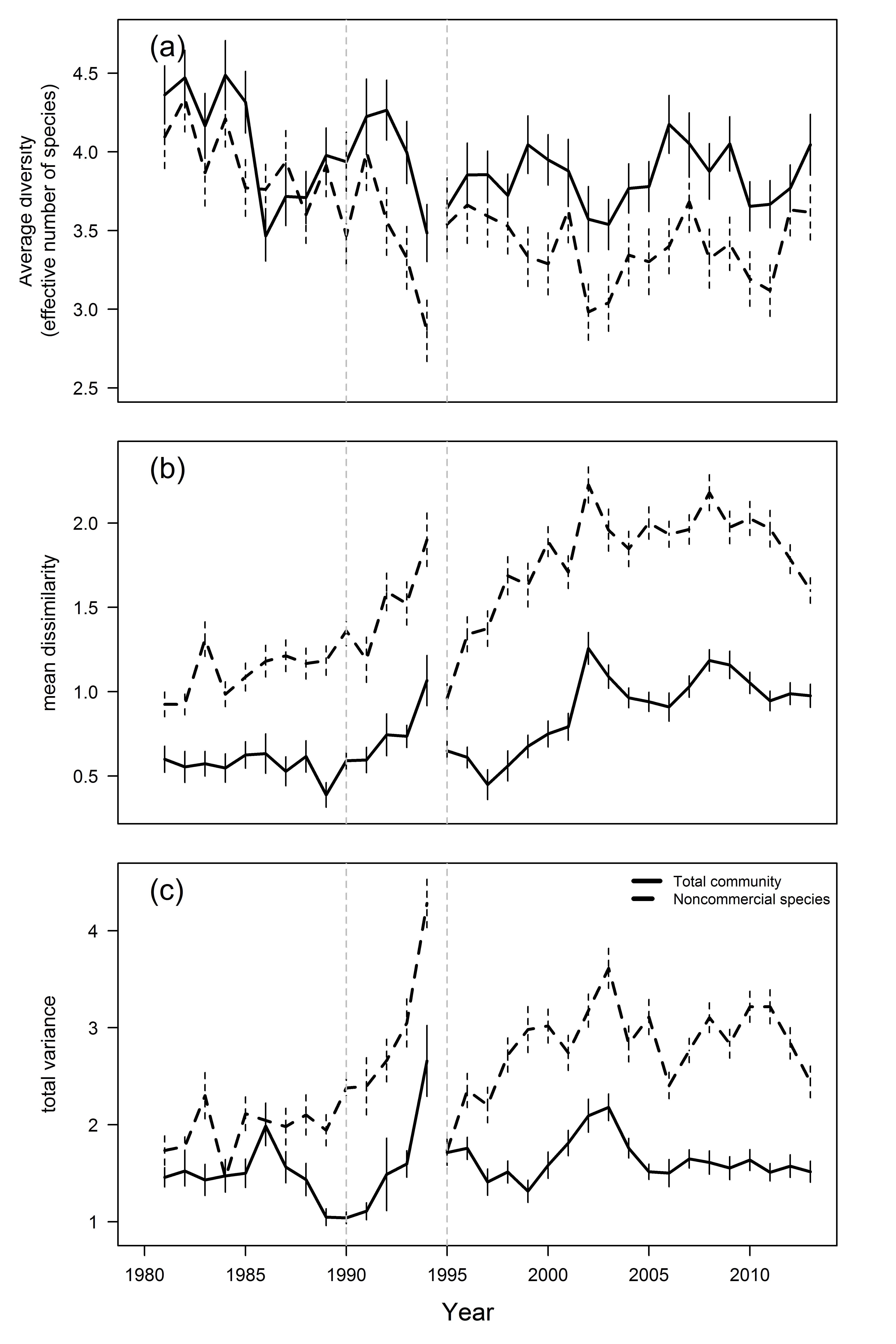


**Figure S4**. Trends in alpha and beta diversity across the study period. (a) Average effective number of species (the exponent of Shannon diversity) per polygon for a given year for all species (solid line) and rare species only (dashed line). Vertical lines indicate +/- 1 standard error. (b) Average pairwise Bray-Curtis dissimilarity between all polygons observed in a given year. Vertical lines indicate jackknife se values. (c) The variance of between-polygon dissimilarity in each year. Lines are as in (b).

## Estimating gear change effects on community dynamics

To deal with the gear change in 1995 from an Engles bottom trawl to a Campelen bottom trawl, in our analysis, we filtered out species whose biomass catches were highly sensitive to the change in gear. As the Campelen trawl had a much smaller mesh size than the Engles, it is expected that smaller fish of all species would be caught after the gear change, increasing estimates of biomass for all species. Unfortunately, estimating this effect is difficult because no comparative fishing with the two gears was conducted in this study region. Furthermore, the gear change occurred during a period of rapid ecological change, meaning that average fish biomass for most species was likely changing during that period.

To determine how sensitive our results might be to this gear change, we estimated multiplicative gear change conversion factors, assuming that the average biomass *B* we would expect to catch of species *i* following the gear change is some factor *q* of the biomass of species *i* prior to the gear change:

$$B_{i,post}=q_{i}B_{i,. pre}$$

To minimize bias in estimating $q_{i}$stemming from changing population sizes, we only used trawls from the years 1993 to 1996 (the two years before and after the gear change). To find comparable trawls before and after the gear change, we *z*-score transformed latitude, longitude, and ln(Depth) across all trawls, then for each trawl from 1995 and 1996 (postchange), we found the nearest neighbouring trawl (measured in Euclidian distance in scaled lat, long, and depth) from the prechange period. For each pair of trawls *j* for each species *i*, we added 0.01 kg to all species biomasses (to account for zero-valued catches) and calculated the ratio of biomass found in the trawl after the gear change ($b_{i,j, post}$) to the biomass found before the gear change ($b_{i,j, pre}$) as $r_{i,j}=\frac{b_{i,j, post}}{b_{i,j, pre}}$. For each species, we discarded any matched trawls where biomass was zero in both pre- and postchange trawls, as these would bias estimates of $q_{i}$ toward one.

We used the R package lme4 to estimate a random effects model to determine conversion factors for all species, by modelling log($r_{i,j})$ as the sum of a species-specific intercept ($ln(q_{i})$) and a normally distributed error. Species specific intercepts were treated as random effects to pool them towards a common mean and to reduce estimation errors in rare species. The full model used was:

$$r_{i,j} =\ln\left( q_{i} \right)+\epsilon_{i,j}$$

$$\ln\left( q_{i} \right)\sim Normal\left( \mu, \sigma_{species} \right)$$

$$\epsilon_{i,j}\sim Normal\left( 0, \sigma_{error} \right)$$

Where the overall mean log conversion rate $\mu$, the random effect variance $\sigma_{species}$, and the observation error $\sigma_{error}$ are hierarchical parameters estimated by lme4 using restricted marginal likelihood (REML).

Estimated conversion factors ($\pm$2 SE) are shown in figure S5. Conversion factors ranged from 0.2 to 10, with all but three species having conversion factors greater than one (as expected). There was also substantial estimation error for many species; in some cases, approximate 95% Cis on conversion factors spanned an order of magnitude.


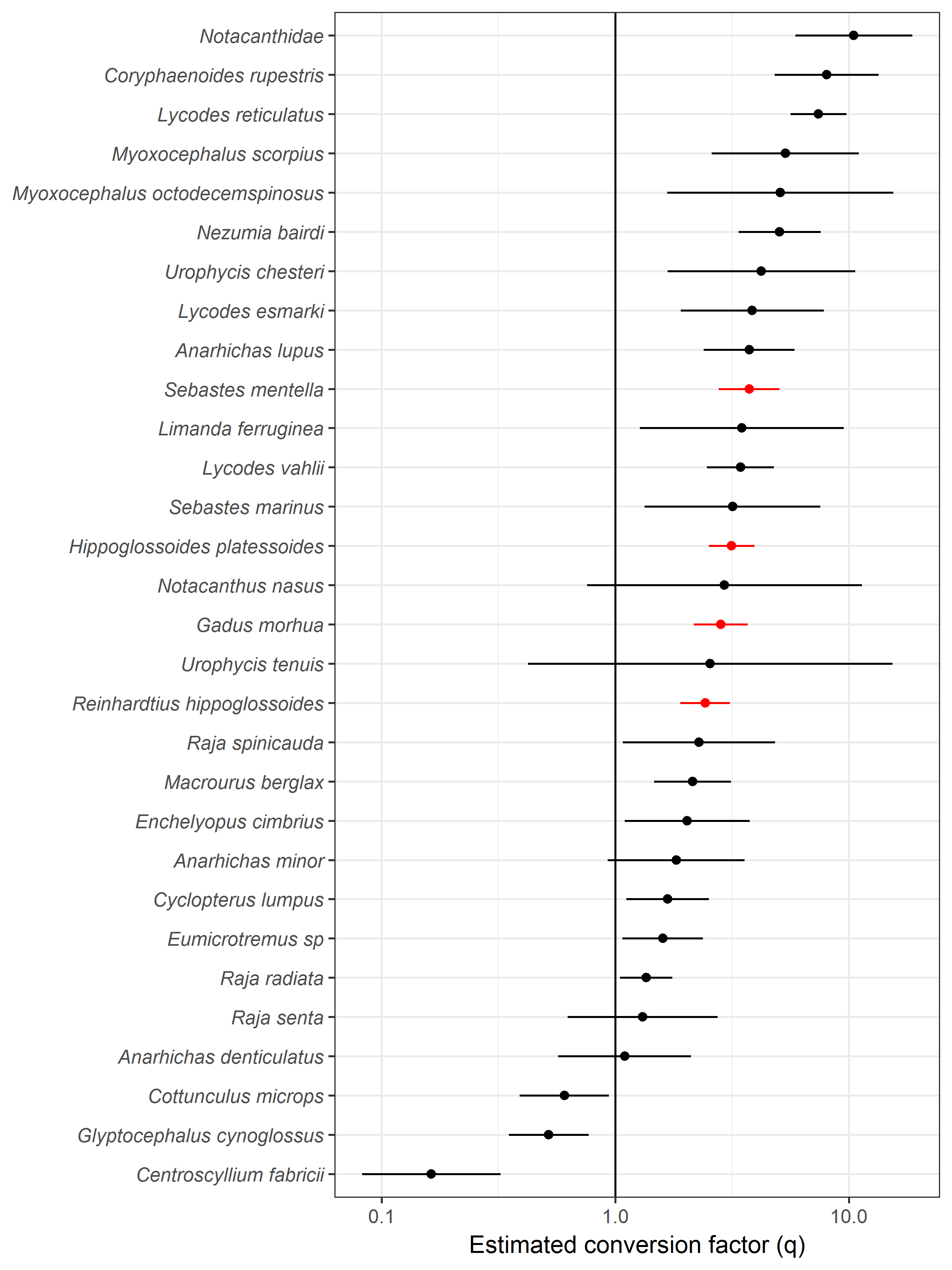


Figure S5: Estimated conversion factors for species in the study (points) with +/- 2 standard error range indicated by horizontal lines. Vertical solid line indicates a conversion factor of 1 (implying the gear change would not affect average catches of that species). The top four species by biomass are highlighted in red.

To determine what affect using conversion factors might have on our results, we multiplied the biomass measures for each species in all trawls after the gear change by 1/$q_{i}$ to scale them to the same level as the pre-gear change biomass measures, the repeated the analyses from the main text. Overall, accounting for conversion factors reduced the estimated degree to which community biomass in both the entire community and the non-commercial community has recovered (figure S6a), as well as in the top four commercial species (figure S6b). However, the community showed a very similar pattern of collapse and recovery of functional diversity (figure S6c) and community composition after accounting for the gear change in both the total groundfish community (figure S6d) and the non-commercial fish community (figure S6e).

We chose not to incorporate these conversion factors into the main analyses in the paper because none of our conclusions changed substantially with the addition of conversion factors. Furthermore, there are still unknown biases missing from our estimated conversion factors (for instance, any change in the true biomass of a given species between 1993/94 and 1995/96 would bias the conversion factor in the direction of the rate of change; using these conversion factors would introduce additional variability into biomass estimates). However, we note that this analysis implies that our results overstate the extent of biomass recovery in these stocks.


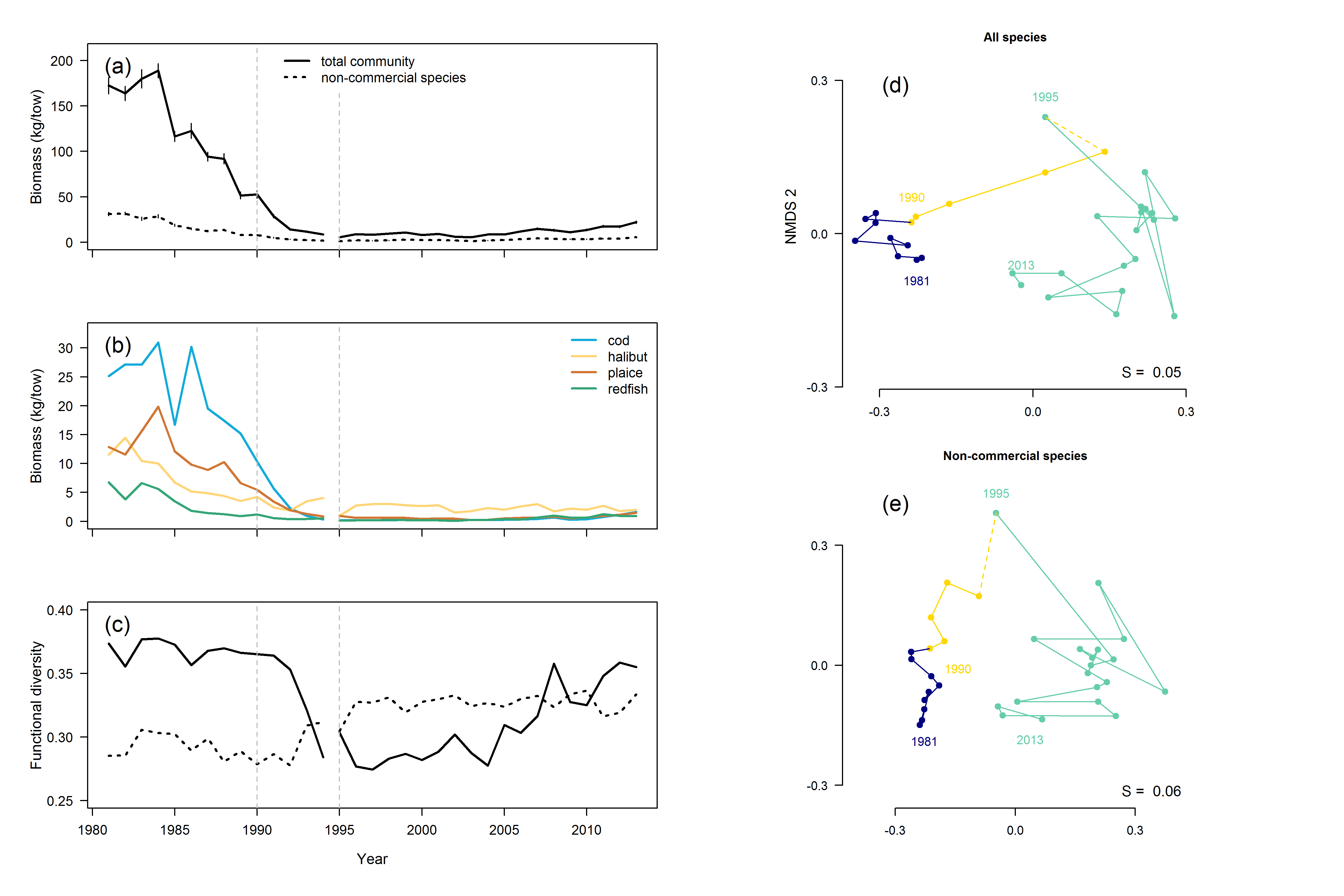


Figure S6. Biomass and compositional dynamics accounting for estimated conversion factors. (a) Mean biomass of the whole community (solid lines) and non-commercial species (dashed lines). Vertical dashed lines represent the collapse in 1990 and the gear change in 1995. (b) Mean biomass trends of the top four commercial species throughout the study period. Vertical dashed lines are as in (a). (c) Trends in functional diversity. Lines are as in (a). (d) NMDS plot of changes in community composition in the entire community. Colors represent three periods: purple: prior to the collapse (1981 – 1989), yellow – the period of the collapse until the gear change (1990 – 1994) and teal – following the gear change (1995-2013). (e) NMDS plots of changes in community composition of the non-commercial species. Colours are as in (d).
